# High prevalence of HIV, HBsAg and anti-HCV positivity among people who injected drugs: results of the first bio-behavioral survey using respondent-driven sampling in two urban areas in Mozambique

**DOI:** 10.1186/s12879-019-4655-2

**Published:** 2019-12-02

**Authors:** Cynthia Semá Baltazar, Roberta Horth, Makini Boothe, Isabel Sathane, Peter Young, Denise Chitsondzo Langa, Manuel Condula, Helena Ricardo, Liliana Dengo Baloi, Beverley Cummings, Nicolas Schaad, Lídia Gouveia, Eugénia Teodoro, Henry F. Raymond

**Affiliations:** 1 National Institute of Health, P.O. Box 264, Maputo, Mozambique; 2 Ghent University, Faculty of Medicine and Health Sciences, Ghent, Belgium; 3 University of California, San Francisco, California, USA; 4 University of California, Global Programs for Research and Training, Maputo, Mozambique; 5 Centers for Disease Control and Prevention, Nairobi, Kenya; 6 Rede Nacional Contra a Droga (UNIDOS), Maputo, Mozambique; 7 Substance Abuse and Mental Health Services Administration, Pretoria, South Africa; 8 Centers for Disease Control and Prevention, Atlanta, GA USA; 9 Department of Mental Health, National Public Health Directorate, Ministry of Health, Maputo, Mozambique; 10 School of Public Health, Rutgers University, Piscataway, NJ USA

**Keywords:** Prevalence, Human immunodeficiency virus, Hepatitis B, Hepatitis C, People who inject drugs, Respondent-driven sampling, Mozambique

## Abstract

**Background:**

Few countries in sub-Saharan Africa know the magnitude of their HIV epidemic among people who inject drugs (PWID). This was the first study in Mozambique to measure prevalence of HIV, HBV, and HCV, and to assess demographic characteristics and risk behaviors in this key population.

**Methods:**

We used respondent-driven sampling (RDS) to conduct a cross-sectional behavioral surveillance survey of PWID in two cities of Mozambique lasting six months. Participants were persons who had ever injected drugs without a prescription. Participants completed a behavioral questionnaire and provided blood specimens for HIV, hepatitis B surface antigen (HBsAg) and hepatitis C virus antibody (anti-HCV) testing. We performed RDS-adjusted analysis in R 3.2 using RDSAT 7.1 weights.

**Results:**

We enrolled 353 PWID in Maputo and 139 in Nampula/Nacala; approximately 95% of participants were men. Disease prevalence in Maputo and Nampula/Nacala, respectively, was 50.1 and 19.9% for HIV, 32.1 and 36.4% for HBsAg positivity, and 44.6 and 7.0% for anti-HCV positivity. Additionally, 8% (Maputo) and 28.6% (Nampula/Nacala) of PWID reported having a genital sore or ulcer in the 12 months preceding the survey. Among PWID who injected drugs in the last month, 50.3% (Maputo) and 49.6% (Nampula/Nacala) shared a needle at least once that month. Condomless sex in the last 12 months was reported by 52.4% of PWID in Maputo and 29.1% in Nampula/Nacala. Among PWID, 31.6% (Maputo) and 41.0% (Nampula/Nacala) had never tested for HIV. In multivariable analysis, PWID who used heroin had 4.3 (Maputo; 95% confidence interval [CI]: 1.2, 18.2) and 2.3 (Nampula/Nacala; 95% CI: 1.2, 4.9) greater odds of having HIV.

**Conclusion:**

Unsafe sexual behaviors and injection practices are frequent among PWID in Mozambique, and likely contribute to the disproportionate burden of disease we found. Intensified efforts in prevention, care, and treatment specific for PWID have the potential to limit disease transmission.

## Background

People who inject drugs (PWID) are at high risk of acquiring and transmitting blood-borne viruses, including HIV, from unsafe injection practices and risky sexual behaviors. The United Nations Office on Drugs and Crime (UNODC) estimates that around 29.5 million people suffer from drug use disorders worldwide [1] of the estimated 15.6 million PWID in the world, sub-Saharan Africa has 1.4 million. The Southern and Eastern coasts of Africa are important routes for global heroin trafficking originating from Asia, and the region is fast becoming a final destination for these drugs, with a growing number of African consumers [2–4].

HIV prevalence among PWID is 18% globally [3], nearly 28 times higher than HIV prevalence among the general adult population [5]. A systematic review determined that HIV prevalence among PWID in sub-Saharan Africa was 56% [3], but estimates in the region vary widely [3].

Adverse health outcomes among PWID are further intensified by punitive legal environments, a variety of human rights abuses and poor access to services [6, 7]. Few harm reduction programs and PWID-specific HIV prevention, care and treatment services are available in sub-Saharan Africa [8–10]. As of 2017, only seven countries had publicly funded syringe exchange and medication-assisted opioid treatment programs [11]. In Mozambique, publicly funded harm reduction and HIV care and treatment programs for PWID did not exist prior to this study.

An estimated 13.2% of the adult population in Mozambique are living with HIV [12–16]; the percentage of PWID living with HIV was unknown. Information on blood-borne disease burden and demographic and behavioral characteristics of PWID are necessary to assess the unique prevention, care and treatment needs of this population. For this purpose, we conducted the first biological and behavioral survey (BBS) among PWID in two urban areas of Mozambique. PWID are one of five priority populations surveyed as part of the national HIV behavioral surveillance system, which include men that have sex with men, female sex workers, long-distance truck drivers, and mine workers [17–20]. This manuscript presents the main findings of the PWID BBS survey.

## Methods

### Formative assessment

Prior to conducing the PWID BBS, we conducted formative assessments consisting of 7 focus groups and 22 key informant interviews with 86 participants in Maputo and Nampula/Nacala in July 2013 to identify the feasibility of using respondent-driven sampling for reaching PWID in the survey cities. In each city, community outreach workers collaborated with a community liaison coordinator, a survey supervisor and survey interviewers to design a participant recruitment plan for formative assessment. These assessments answered operational and logistical questions related to survey implementation, and obtained information from PWID and providers, such as locations of injection use and availability of health and social services. Formative assessment found that RDS would be a feasible methodology, that PWID were highly networked from diverse social background, and that most PWID were men. In terms of risk behaviors, needle sharing was a common practice, and that there was little access to harm reduction service. In terms of study operation, formative assessment determined that incentives to help cover cost of transport and time would be helpful, fingerprick was preferable to venous blood draw, participants would be willing to test for HIV, HBV and HCV [21].

### Study setting

From October 2013 to March 2014, we conducted a cross-sectional biobehavioral survey using respondent-driven sampling (RDS) methodology in Maputo and Nampula/Nacala. Maputo, the capital city, is located in the South and is the most populous city in the country, while Nampula, located in the North, and the second most population city. These was selected as survey cities as a result of stakeholder consultations that assessed programmatic data, civil society organizations that reported a large number of PWID in the area. Both are also important port cities for the trafficking of heroin from Asia to South Africa and eventually Europe [4]. A satellite site was established in Nacala city, 200 km from Nampula because PWID in that city shared the same social network as those in Nampula. Survey sites were private venues rented for the purpose of the survey. Access to sites was restricted to survey teams and to persons with a survey coupon. Survey sites were staffed by a supervisor, a coupon manager, a receptionist, three interviewers, three testing counselors, and a security guard. Survey teams received two weeks of training on PWID, human subjects research, and RDS. The training comprises theoretical presentations as well as survey simulations, facilitated by the survey investigators.

### Participant eligibility

Persons were eligible to participate if they were 18 years of age or older and lived, worked, or socialized in the survey area during the six months preceding the survey, were in possession of a valid referral coupon, and had not previously participated in the current round of the study. Additionally, persons who participated in the survey prior to October 2013 must have injected drugs without a prescription in the 12 months preceding the survey. Due to bottlenecks in social networks, starting in December 2013, we received ethical review board approval to alter expand our inclusion criteria to include any person who had ever injected drugs without a prescription. During participant eligibility screening, community outreach workers, current or prior injection drug users themselves, further assisted interviewers in determining participant eligibility by assessing whether or not participants were familiar with the local injection drug use culture.

### Participant recruitment and sample size

RDS combines non-probability snowball sampling with mathematical modeling to weight a non-randomly selected sample to generate probability-based population estimates [22]. Participant recruitment began with ‘seeds’ (initial participants) who were purposively selected in consultation with key informants within the PWID community. All seeds were selected before the study began, but only three seeds were given coupons at the beginning of the study at each site. In Nampula, two additional seeds were given coupons one month after initiation, and two more the month after. In Maputo, two seeds were given coupons two months after initiation. In order to cover different subgroups of the target population, seeds were selected based on diverse characteristics including sex, age, education and neighborhood of drug purchase or consumption. Additionally, seeds had large social network sizes and were able to recruit peers. Each seed received three to five recruitment coupons to recruit peers after participating in the study. Their peers who then participated in the survey were also given three to five coupons to recruit their peers. Coupons received a brief training given the coupon manager on how to recruit their peers and an information sheet explaining the process. Coupons were tracked using RDSCM, v 3.0 and on a manual log book.

This process was repeated with subsequent recruits until the sample size was met and/or equilibrium was achieved. To ensure that participants did not participate more than once, there was only one survey site per city staffed by the same people during opening hours, community outreach workers who were current injectors were trained to be aware of who had already participated, and the coupon manager would note specific participant markings (e.g. tattoos and scars). The target sample size was set at 350 in each city based on the ability to provide 5% absolute precision around an estimated prevalence of 15% with a design effect of 2.3. The only available estimate of HIV among PWID in Mozambique came from survey of incarcerated persons which found prevalence of 32.1% in 2006 [23]. We estimated prevalence among non-incarcerated populations of PWID to be about half that of incarcerated persons’. Equilibrium is the point at which the RDS sample proportions for key variables no longer change (or change very minimally) no matter how many more people are recruited; equilibrium was monitored weekly throughout survey implementation. In Nampula/Nacala the desired sample size was not met, although efforts were made to improve recruitment, such as by improving outreach and education using community outreach workers who were themselves PWID and by increasing the number of coupons given to each participant to five. Although the sample size was lower than anticipated, we assessed equilibrium for key variables, including age, HIV status, length of time of drug use, drug of choice, to ensure that these proportions had stabilized over enrollment.

Participants received a prevention and hygiene kit, which included toothpaste, toothbrush, razor, band aids, alcohol swabs, soap, condoms, lubricants and health information, for participating in the survey. They also received 50 MZN (~US$2.50 at the time of survey) in cash for compensation of transportation costs. Participants received a mobile phone card worth 50 MZN for each referred peer who completed the survey. Each participant could refer up to five peers.

### Behavioral questionnaire

Behavioral data were collected via a structured interview using Questionnaire Development System (QDS™) version 2.6.1 (NOVA Research Company, Silver Spring, MD). The survey tool included questions related to participant socio-demographic characteristics, sexual and injection risk practices potentially related to HIV, HBV, HCV infections and other sexually-transmitted infections (STI), violence and discrimination, and utilization of health services. Individual social network sizes, required for RDS analyses, were measured by asking “Approximately how many people who inject drugs do you personally know by name who also know your name?” The survey tool was developed in Portuguese and English and offered in both languages. Tools were pre-tested with PWID.

### Biological sample collection and testing

Participants were offered HIV counseling and testing consistent with national HIV testing guidelines using a serial algorithm that screens with Determine™ HIV-1/2 (Alere, USA), and confirms with Uni-Gold *HIV*™ (Trinity Biotech, Ireland) [24]. Following national norms, participants reporting a prior HIV positive test result were not retested during this study. HIV prevalence estimates were based on a combination of HIV rapid test results and self-reported seropositivity. All blood used for point-of-care tests and for preparation of Whatman™ 903 filter paper were collected by a single finger prick from consenting participants. Dried blood spots were used for external quality control, and 2% of the negative samples and 5% of the randomly positive samples were sent to the regional reference laboratory for HIV testing (National Institute for Communicable Diseases in South Africa Republic); concordance was 97.7%.

Point-of-care testing for HBsAg (Alere Determine® HbsAg, Abbott Laboratories, UK) and anti-HCV (SD Bioline HCV, Standard Diagnostics, Korea), regardless of prior testing history, was provided during the survey. While a reactive HBsAg test indicates the person is currently infected with either acute or chronic hepatitis B, a reactive anti-HCV test indicates either current viremia or a past HCV infection that resolved naturally or was cured with treatment. Central hepatitis B tests were performed using ELISA Murex® HBsAg Version 3 (Murex Biotech Limited, UK) at the National Institute of Health Laboratory, following internal quality controls, and was used to estimate prevalence in the survey; centralized anti-HCV testing was not performed.

A unique participant sequential code was used to link test results to survey data. All point-of-care test results were returned to participants promptly after test completion. Centralized testing results were not returned as these tests were for quality control purposes only. All participants who tested positive on any point-of-care test received counseling and referrals for follow-up care within the national health system using referral coupons.

Participants who had their coupons stamped at the referral health clinic were reimbursed the value of public transportation to the clinic (50 MZN or approximately US$2.50). Hepatitis C treatment was not available in the country at the time of the study.

### Data analysis

Diagnostic procedures, including analysis of homophily and convergence, were run to ensure that data met the underlying assumptions of RDS. Population point prevalence estimates and bootstrapped 95% confidence intervals, shown in Tables 1, 2, 3, and 4, were produced using the RDS-1 enhanced data smoothing estimator in RDSAT 7.1 (Cornell University, Ithaca, NY). In RDSAT, the number of re-samples for bootstrap was set to 15,000 and the algorithm type as “enhanced data-smoothing” as recommended by software developers [17]. Seeds were included in analysis. A participant was considered to be sexually active if he or she reported having had one or more sexual partners in the last 12 months. Participants reporting no sexual partners in the last 12 months were not asked questions related to recent sexual behaviors. The outcome measure of interest presented in this analysis is HIV status. All individuals with a positive on-site HIV test and those who reported knowing their HIV positive status who did not test on site were classified as having HIV. Individuals who tested negative or had an indeterminate result were classified as negative. Individuals who refused testing and did not report having had a prior HIV positive test result were classified as missing. Design effect for the outcome of interest was 2.6 for Maputo and 1.5 for Nampula. Sensitivity analysis was performed classifying individuals with missing status as positive and negative.

**Table 1:** RDS-adjusted point estimates and confidence intervals of socio-demographic characteristics of people who inject drugs in Mozambique, 2013–2014

	Maputo (*n* = 353)				Nampula/Nacala (*n* = 139)			
	n^a^	Crude	Adj ^b^ %	95% CI	n^a^	Crude	Adj ^b^ %	95% CI
Sex								
Male (vs Female)	332	94.1	92.9	87.8–97.1	135	97.1	97.0	92.6–100
Age (Years), median [IQR]^c^		33 [19, 56]				28 [18, 60]		
18–24	37	10.5	12.5	7.7–17.0	55	39.6	42.6	30.1–56.7
25–34	179	50.7	54.4	47.4–60.6	50	36.0	34.5	24.1–45.3
≥ 35	137	38.8	33.1	27.6–40.6	34	24.5	22.9	12.0–34.4
Marital status								
Single (never married)	226	64.0	65.4	58.7–72.1	63	45.3	49.7	38.4–61.8
Married or in a conjugal union	55	15.6	16.7	10.9–23.1	48	34.5	32.1	21.7–43.5
Separated, widowed or divorced	72	20.4	17.8	13.1–23.0	28	20.1	18.3	9.6–27.6
Education								
Primary and below	181	51. 4	57.0	49.5–64.0	29	20.9	28.4	16.5–40.6
Missing	1	–	–		–	–	–	
Primary language spoken at home								
Portuguese (vs Other)	270	76.5	71.4	64.4–78.3	105	75.5	67.6	55.1–79.7
Nationality								
Mozambican (vs Other)	351	99.4	99.8	99.4–100	132	95.0	96.7	93.2–99.3

^a^ Sample includes seeds; no missing responses unless specified

^b^ Estimates and 95% confidence intervals (CI) adjusted for RDS survey design using RDS-I (DS) estimator produced with RDSAT 7.1

^c^ IQR Interquartile range

**Table 2:** RDS-adjusted point estimates and confidence intervals of behavioral characteristics related to drug consumption among people who inject drugs in Mozambique, 2013–2014

	Maputo (*n* = 353)				Nampula/Nacala (*n* = 139)			
	n^a^	Crude	Adj^b^ %	95% CI	n^a^	Crude	Adj^b^ %	95% CI
Age (in years) of first injection drug use, median [IQR]		24 [9, 46]				20 [14, 47]		
< 18	37	10.9	7.0	4.5–10.7	19	13.7	9.6	4.6–15.0
18–24	152	44.7	48.8	41.9–55.4	83	59.7	60.1	50.5–70.3
≥ 25	151	44.4	44.2	36.9–50.8	37	26.6	30.3	19.8–40.7
Missing	13	–	–		0	–	–	
Years since injection initiation, median [IQR]		7 [0, 35]				5 [0, 29]		
0	11	3.2	4.4	1.4–7.7	5	3.6	5.1	1.6–9.6
1–5	122	35.9	41.6	34.5–50.7	72	51.8	52.2	40.9–63.2
6–10	87	25.6	25.1	18.0–30.6	27	19.4	19.8	12.8–29.7
≥ 11	120	35.3	28.9	22.7–36.0	35	25.2	22.9	12.2–33.0
Missing	13	–	–		0	–	–	
Drug most commonly injected								
Heroin	305	86.6	82.2	76.0–87.7	105	76.1	73.3	62.0–84.0
Cocaine	47	13.4	17.8	12.3–24.0	18	13.0	14.0	5.4–22.3
Other (including crack and cocktail mixes)	0	0.0	0.0		15	10.9	12.7	6.1–22.5
Missing	1	–	–		1	–	–	
Frequency of injection drug use, last 12 months								
Did not inject, last 12 months	18	5.1	8.6	3.9–13.9	7	5.0	3.8	0.3–8.1
A few times per year	17	4.8	5.0	2.2–9.0	12	8.6	10.9	4.1–19.3
A few times per month	39	11.0	10.8	6.9–15.0	31	22.3	33.5	22.4–46.7
A few times per week	39	11.0	13.6	9.0–19.1	54	38.8	32.4	23.2–40.6
Daily	240	68.0	62.0	54.4–69.4	35	25.2	19.4	11.3–29.5
Ever shared a syringe or needle, lifetime								
Yes (vs No)	189	53.5	50.3	43.0–57.6	69	49.6	42.4	31.0–55.0
Injected drugs, last month								
Yes (vs No)	267	75.9	67.5	58.6–76.7	124	89.2	92.8	87.9–98.0
Missing	1	–	–		0	–	–	
Number of people with whom shared needles or syringes, last month								
0	248	70.3	74.1	67.6–79.6	98	70.5	70.8	61.8–80.2
1–2	26	7.4	6.8	3.9–10.5	24	17.3	18.3	9.7–26.5
≥ 3	55	15.6	14.1	9.6–19.3	17	12.2	11.0	5.5–17.4
Shared, but doesn’t remember with how many	24	6.8	5.1	2.4–8.4	0			

^a^ Sample includes seeds; no missing responses unless specified

^b^ Adjusted estimates and 95% confidence intervals (CI) produced with RDSAT 7.1

**Table 3:** RDS-adjusted point estimates and confidence intervals of sexual risk behaviors among people who inject drugs, Mozambique 2013–2014

	Maputo (*n* = 353)				Nampula/Nacala (*n* = 139)			
	n^a^	Crude	Adj ^b^ %	95% CI	n^a^	Crude	Adj ^b^ %	95% CI
Number of sexual partners, last 12 months^c^								
0	93	27.2	24.4	18.8–30.7	5	3.6	3.0	0.4–6.3
1	86	25.1	27.9	21.6–35.5	18	12.9	16.2	6.7–27.6
≥ 2	163	47.7	47.7	39.1–55.4	116	83.5	80.8	69.3–91.1
Missing^d^	11	–	–		–	–	–	
Ever had sex in exchange for drugs, lifetime								
Yes (vs No)	47	13.7	11.4	7.3–15.9	20	14.4	10.3	4.8–16.2
Missing^d^	11	–	–		0	–	–	
Gave money, goods or services in exchange for sex, last 12 months								
Yes (vs No)	114	33.3	30.3	24.5–37.2	89	64.0	65.7	55.2–76.2
Missing^d^	11	–	–		0	–	–	
Received money, goods or services in exchange for sex, last 12 months								
Yes (vs No)	60	17.5	17.5	12.2–22.6	42	30.2	27.3	17.5–38.1
Missing^d^	11	–	–		0	–	–	
Had unprotected intercourse at last sex, last 12 months^d,e^								
Yes (vs No)	131	52.0	52.4	44.8–61.6	42	31.1	29.1	19.3–40.3
Missing^d^	1	–	–		0	–	–	
Last sexual partner injects drugs, last 12 months^d,e^								
Yes (vs No)	31	12.5	10.0	5.7–15.9	8	6.0	4.6	1.5–8.8
Missing^d^	5	–	–		1	–	–	
Shared a needle or syringe with last sex partner, last 12 months^d,e^								
Yes (vs No)	14	5.6	4.5	1.8–85.0	3	2.2	2.0	0.0–5.1
Missing^d^	5				1			

^a^ Sample includes seeds; no missing responses unless specified

^b^ Adjusted estimates and 95% confidence intervals (CI) produced with RDSAT 7.1

^c^ Includes male and female partners of men that inject drugs and male partners of women that inject drugs

^d^ Missing responses include those that had sexual partners and could not remember or refused to answer to the question

^e^ Sub-analysis of persons who had sex in past 12 months in Maputo (*n* = 253) and Nampula/Nacala (*n* = 135)

**Table 4:** RDS-adjusted point estimates and confidence intervals of health outcomes and service utilization among people who inject drugs in Mozambique, 2013–2014

	Maputo (*n* = 353)				Nampula/Nacala (*n* = 139)			
	n^a^	Crude	Adj ^b^ %	95% CI	n^a^	Crude	Adj ^b^ %	95% CI
Has access to new, unused syringes or needles	315	89.2	89.6	85.4–93.6	110	79.7	78.9	69.6–87.2
Missing	0	–	–		1	–	–	
Had contact with a peer educator, last 12 months	40	11.4	9.5	5.3–13.3	62	44.6	40.8	31.5–52.0
Missing	1	–	–		0	–	–	
Received free condoms, last 12 months	100	28.6	26.8	21.0–33.7	88	63.3	60.0	49.2–71.3
Missing	3	–	–		0	–	–	
Ever received treatment for overdose, drug substitution therapy or detoxification, lifetime	16	4.5	2.5	1.2–4.1	37	26.6	23.7	16.4–31.9
Ever in a rehabilitation or a self-help group, lifetime^c^	12	3.4	2.5	0.9–4.8	26	18.7	14.3	7.8–22.1
Ever incarcerated, lifetime	264	74.8	72.2	66.4–78.0	64	46.0	42.6	29.9–52.9
Previous HIV test, lifetime	254	72.0	68.4	61.1–75.4	80	57.6	59.0	46.4–68.6
Previous HIV test, last 12 months	80	22.9	24.6	18.9–30.0	35	25.2	21.9	12.7–31.4
Missing	4	–	–	–	0	–	–	
Ever heard of Hepatitis B or C	138	39.1	34.7	28.3–41.4	73	52.5	52.1	40.8–617
Has HIV	179	56.1	50.1	40.1–59.0	25	19.8	19.9	10.9–29.2
Did not consent	34				13			
Screen positive for Hepatitis B surface antigen	107	30.5	32.1	25.2–38.5	57	41.0	36.4	22.6–49.8
Did not consent	2				0			
Hepatitis C antibodies	148	47.6	44.6	33.4–53.9	15	11.1	7.0	2.0–12.5
Did not consent	42				4			
Has HIV & HBV surface antigen	49	15.4	14.9	9.1–19.5	10	7.9	8.3	2.4–14.9
Did not consent	35				13			
Has HIV & HCV antibodies	116	38.7	36.1	26.4–45.8	8	6.5	4.1	0.6–8.4
Did not consent	53				15			
Ever tested positive for HIV prior to the survey	123	34.9	29.0	22.1–44.3	10	7.2	5.8	1.7–11.2
Missing	1				1			
Had a self-reported sore or ulcer in the genital region, last 12 months	30	8.5	8.0	4.7–11.7	35	25.2	28.6	19.1–37.9

^a^ Sample includes seeds; no missing responses unless specified

^b^ Adjusted estimates and 95% confidence intervals (CI) produced with RDSAT 7.1

^c^ Includes self-help groups, outpatient counseling, drug substitution therapy, detoxification program or rehabilitation

Individual weights based on the outcome of interest (HIV status) were generated in RDSAT and exported to R to be used in bivariable and multivariable analysis (presented in Table 5). Logistic regression, using the *svyglm* command for complex weighted surveys, was used to model associations with HIV status. Data-driven categorization was used for continuous variables. Variables considered for modeling (listed in Tables 1, 2, 3, and 4) were selected a priori based on evidence in the literature about associations with HIV and potential confounders. Those that in bivariable analysis were significant at *P* < 0.2 in any of the two cities, those that are known confounders (i.e., education and gender) and those presenting unexpected outcomes (i.e., prior incarceration) were then selected for multivariable modeling. Missing values were excluded from analysis; and variables related to most recent sexual partner were not considered in analysis because only participants with a sexual partner in the last 12 months were asked these questions. Interaction effects and multicollinearity between variables were ascertained during modeling. Several models were developed, and the Akaike Information Criteria was used to systematically remove variables.

**Table 5:** RDSAT-weighted bivariable and multivariable associations of demographic and behavioral characteristics associated with HIV among PWID, Mozambique 2013–2014

Variables	Maputo						Nampula/Nacala					
	OR^a^	95% CI	*p*-value	aOR^a,b^	95% CI	*p*-value	OR^b^	95% CI	*p*-value	aOR^a,c^	95% CI	*p*-value
	*n* = 351			*n* = 333			*n* = 139			*n* = 137		
Ages 35+ (ref: 18–34 years)	1.5	0.9–2.3	0.09				3.7	1.5–9.4	< 0.01	9.3	1.9–65.8	0.01
Years since first injected (ref: 0–5 years)												
6–10 years	2.0	1.1–3.5	0.02	2.0	1.1–3.8	0.03	16.6	5.1–62.8	< 0.01	22.8	5.3–126.4	< 0.01
11 or more years	2.3	1.4–4.0	< 0.01	2.1	1.2–3.8	0.01	4.2	1.2–15.2	0.02	1.6	0.2–10.2	0.62
Marital status (ref: single; never married)												
Married or in a conjugal union	0.8	0.4–1.4	0.45				2.2	0.8–6.0	0.11			
Separated, widowed or divorced	1.3	0.7–2.3	0.42				1.1	0.3–3.9	0.84			
Some secondary or higher (ref: primary or less)	1.0	0.6–1.6	0.99				1.3	0.5–3.8	0.58			
Spoke Portuguese as the primary language at home (ref: other language)	1.2	0.7–2.0	0.45				0.8	0.3–2.0	0.59			
Choice of injection drug is heroin (ref: cocaine or other)	2.4	1.3–4.6	0.01	2.3	1.2–4.9	0.02	1.7	0.6–5.5	0.35	4.3	1.2–18.2	0.03
Ever shared a syringe or needle, lifetime	3.4	2.2–5.5	< 0.01	3.2	1.9–5.3	< 0.01	1.5	0.6–3.7	0.35			
Ever incarcerated	1.6	1.0–2.5	0.07				2.0	0.8–5.0	0.12	3.2	1.0–10.9	0.06
Ever had sex in exchange for drugs, lifetime	0.6	0.3–1.2	0.16	0.5	0.2–1.0	0.05	1.3	0.3–5.0	0.70			
Did not have access to new, unused syringes or needles	2.9	1.3–7.1	0.01				0.1	0.0–0.6	0.04	0.1	< 0.01–0.4	0.02
Injected daily, last thirty days	2.0	1.3–3.2	< 0.01	2.0	1.2–3.4	0.01	3.4	1.3–8.9	0.01			
Ever tested for HIV test, lifetime	2.2	1.3–3.7	< 0.01	2.3	1.3–4.0	< 0.01	2.8	1.1–8.2	0.04			

^a^ Weights imported from RDSAT 7.1

^b^ Adjusted for all other variables in the model, LL − 177.913 (df = 8)

^c^ Adjusted for all other variables in the model, LL −39.09976 (df = 7)

Note: Variables were retained in the model (aOR) based on theoretical importance; blank cells indicate those variables that were systematically removed using the Akaike Information Criterion (AIC)

### Ethical considerations

Participants were asked to provide written informed consent for each study component: questionnaire, HIV, HBV and HCV testing, and dried-blood spot sample collection. No personal identifying information was collected from participants. All data were collected on encrypted password protected computers and transferred to a secure central database and deleted locally daily.

## Results

### Participant recruitment

**Fig. 1:**
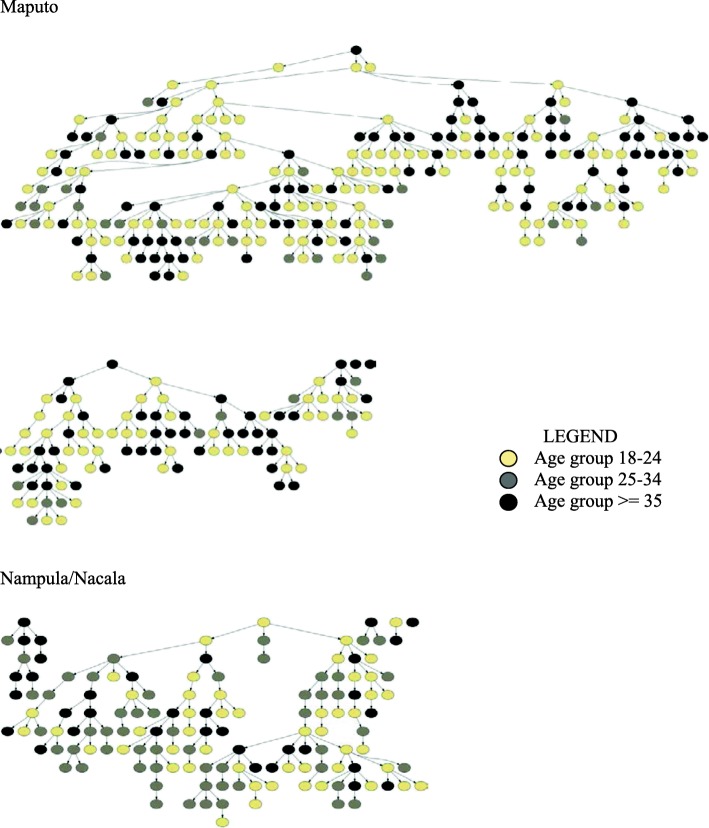
Recruitment networks in Maputo and Nampula/Nacala, BBS-PWID, 2013–2014. Seven seeds were selected in Maputo, of which five participated in the survey and only three seeds recruited participants. The chain of one seed reached 252 participants, the second reached 80 participants, and the third chain reached19 participants. In Maputo the maximum number of recruitment waves was 13. Five seeds were selected in Nampula/Nacala, however, only one seed recruited 123 of the participants in the survey. The maximum number of recruitment waves in Nampula/Nacala was 11. In Maputo, all five seeds that participated in the survey were 35 years old or older and along the processes the participants age become more diversified. In Nampula/Nacala the seed that contributed most in the recruitment of participants was in the 18–24 years old group, and subsequently there was an age diversification

Recruitment took place from October 2013 to March 2014 and lasted approximately 21 weeks in Maputo and 23 weeks in Nampula/Nacala. Five seeds (20% female and 20% > 40 years old) were selected in Maputo and seven in Nampula/Nacala (14% female and 14% > 40 years old). From the five identified seeds in Maputo, only three contributed to recruitment. The longest recruitment chain in Maputo was comprised of 13 waves and 252 participants (71.4% of the total sample). In Nampula/Nacala, seven seeds were identified, of which five participated, and one contributed most substantially to participant recruitment. The maximum number of waves in Nampula/Nacala was 11 with 123 participants (88.5% of the total sample). Figure 1 illustrates the peer-referral recruitment chains of PWID in the two areas of Mozambique. Diagnostic tests revealed that RDS assumptions had been met. Median network size was 15 in Maputo and 10 in Nampula/Nacala.

A total of 1915 and 1210 coupons were distributed in Maputo and Nampula/Nacala (up to five per participant) respectively. Of those, 383 (20%) in Maputo and 241 (20%) in Nampula/Nacala were redeemed by individuals who were screened for eligibility. A total of 353 PWID were enrolled in the survey in Maputo (92% of individuals screened) and 139 in Nampula/Nacala (58% of individuals screened). In both locations, most persons were not eligible because they had not injected drugs in the last 12 months prior to the survey (prior to criteria change). A comparison of sample composition before and after criteria change revealed no significant differences.

### Demographic characteristics of PWID

Most PWID in Maputo and Nampula/Nacala were men (92.9 and 97.0%, respectively) and of Mozambican nationality (99.8 and 96.7%) (Table 1). Median age of PWID in Maputo was 33 years and 28 years in Nampula/Nacala. In Maputo and Nampula/Nacala, 65.4 and 49.7% of PWID, respectively, were single (never married). In Maputo and Nampula/Nacala, 57.0 and 28.4% of PWID, respectively, had a primary education level or below. Portuguese was reported as the primary spoken language among majority of respondents in both survey cities.

### Drug injection behaviors

Many PWID first injected drugs when they were 18–24 years old (48.8% in Maputo and 60.1% in Nampula/Nacala) (Table 2). Close to half (41.6% in Maputo and 52.2% in Nampula/Nacala) began injecting within 1–5 years prior to the survey. In Maputo, the majority of PWID injected drugs daily (62.0%), whereas in Nampula/Nacala 19.4% injected daily.

The most commonly injected drug was heroin in both cities, 82.2% in Maputo and 73.3%, Nampula/Nacala. Close to half (50.3% in Maputo and 42.4% in Nampula/Nacala) of PWID had ever shared a needle or syringe, and 14.1% in Maputo and 11.0% in Nampula/Nacala shared a needle with ≥3 different persons in the last month. In Maputo 14.3% and in Nampula/Nacala 8.0% of PWID used a needle or syringe after someone else had injected with it; and 24.9% (Maputo) and 22.7% (Nampula/Nacala) passed on their used needle or syringe to someone else (data not in table).

### Sexual behaviors

Nearly half (47.7%) of PWID in Maputo, and the majority (83.5%) in Nampula/Nacala, had ≥2 sexual partners in the year preceding the survey (Table 3). One in ten or fewer (10.0% Maputo and 6.0% Maputo/Nacala) of PWID had a sexual partner that also injected drugs. Half (52.4%) of PWID in Maputo, and almost one third (31.1%) in Nampula/Nacala had unprotected intercourse the last time they had sex. In Maputo, 30.3% of PWID gave money, goods, or services in exchange for sex in the last 12 months, and 17.5% received money, good, or services in exchange for sex. In Nampula/Nacala, 65.7 and 27.3%, did so respectively. Eight percent in Maputo and 28.6% of PWID in Nampula/Nacala reported having a genital sore or ulcer in the 12 months preceding the survey.

### Access to programs

The majority of PWID in both Maputo and Nampula/Nacala at 89.6 and 78.9%, respectively had access to new or sterile syringes (Table 4). As far as contact with peer educators, one in ten (9.5%) of PWID in Maputo and 40.8% in Nampula/Nacala had contact with a peer educator in the last 12 months. Just over one-quarter (26.8%) and 60.0% of PWID in Maputo and Nampula/Nacala had received free condoms in the 12 months preceding the survey. In Maputo, 2.5% of PWID had ever received treatment for overdose, drug substitution therapy and detoxification, while in Nampula/Nacala this proportion was 23.7%. Among PWID, 68.4% in Maputo and 59.0% in Nampula/Nacala had been tested for HIV at least once before this survey, and 24.6% in Maputo and 21.9% in Nampula/Nacala had done so in the 12 months before the survey.

### HIV, HBsAg, and anti-HCV positivity prevalence and associated risks

The prevalence of HIV was 50.1% (95% CI: 40.1–59.0) in Maputo and 19.9% (95% CI: 10.9–29.2) in Nampula/Nacala. Of the 179 participants classified as having HIV, 45 reported prior HIV-positive test result and were not retested. In Nampula, of the 25 participants with HIV, five did not test due to previous positive status. Sensitivity analysis which excluded individuals with self-reported positive HIV status from analysis revealed that prevalence estimates were within the confidence bounds of the prevalence estimate when individuals were retained in the analysis. One third (33.5%) of HIV-positive PWID in Maputo and two-thirds (66.0%) in Nampula/Nacala were not aware of their HIV-positive status.

The prevalence of hepatitis B (acute or chronic) among PWID was also high; 32.1% (95% CI: 25.2–38.5) in Maputo and 36.4% (95% CI: 22.6–49.8) in Nampula/Nacala. Anti-HCV positivity prevalence was 44.6% (95% CI: 33.4–53.9) in Maputo and 7.0% (95% CI: 2.0–12.5) in Nampula/Nacala. Co-infection of HIV and HBV was found in 14.9% (95% CI: 9.1–19.5) of PWID in Maputo and 8.3% (95% CI: 2.4–14.9) in Nampula/Nacala, while the prevalence of HIV and anti-HCV positivity was 36.1% (95% CI: 26.4–45.8) in Maputo and 4.1% (95% CI: 0.6–8.4) in Nampula/Nacala.

Multivariable analysis of associations with HIV positivity among PWID in Maputo and Nampula/Nacala (Table 5) found that the odds of having HIV was greater among those injected drugs for the first time 6–10 years prior to the study compared to 0–5 years prior (adjusted odds ratio [aOR]: 2.0 [95% CI: 1.1–3.8] and 22.8 [95% CI: 5.3–126.4], for Maputo and Nampula/Nacala, respectively), and those that used heroin as their primary drug of choice as compared to cocaine or other drugs (aOR: 2.3 [95% CI: 1.2–4.9] and 4.3 [95% CI: 1.2–18.2], for Maputo and Nampula/Nacala, respectively). In Maputo, these odds were also greater among those who ever shared a syringe or needle (aOR: 3.2 [95% CI: 1.9–5.3]), injected daily (aOR: 2.0 [95% CI: 1.2–3.4]) and had ever been tested for HIV (aOR: 2.3 [95% CI: 1.2–4.0]). Those that had ever had sex in exchange for drugs had lower odds of having HIV in Maputo (aOR: 0.5 [95% CI: 0.2–1.0]). In Nampula/Nacala HIV was associated with prior incarceration (aOR: 3.2 [95% CI: 1.0–10.9]). Additionally, no association was detected between HIV status and level of education, marital status or language in either city.

## Discussion

This was the first study to measures burden of blood-borne diseases and risk factors among PWID in Mozambique. We found an alarmingly high prevalence of HIV, HBsAg and anti-HCV among PWID. HIV prevalence among PWID (50.1% [95% CI: 40.1–59.0] in Maputo and 19.9% [95% CI: 10.9–29.2] in Nampula) was nearly four times higher than the prevalence of HIV among adults in Mozambique (13.2% nationally, 16.9% in Maputo, and 5.7% in Nampula Province) [16]. The differences between the prevalence rates in Maputo compared to Nampula are consistent with the regional characteristics of the epidemic in the country, where higher HIV prevalence is concentrated in the southern region of the country given the historical economic and cultural interaction with neighboring high-prevalence countries, such as South Africa and Eswatini [16, 18]. Prevalence of HIV among PWID in our study is substantially higher than in the prevalence of HIV among PWID in other sub-Saharan African countries, including Nigeria (3.1%), Seychelles (3.8%) and Madagascar (4.8) [3]. While the PWID prevalence in Maputo is much higher than estimates from PWID in South Africa (21%) [19], Mauritius (45.5%), Kenya (42.0%), and also similar (but less) than what is estimated in sub-Saharan region (56% in 2017) [3].

This high burden of disease is especially concerning because many of PWID with HIV were also coinfection with HBV and HCV which are known to contribute to worse health outcomes [20]. The proportion of PWID with HBV (as measured by HBsAg) in our study is similar that of PWID in other sub-Saharan countries [3], but it is substantially higher than non-PWID untreated HIV-infected living with HIV in Maputo which estimated the prevalence to be 9.1% [25] and 12.2% among non-PWID young people [14]. This high prevalence may be linked to the fact that the hepatitis B vaccine is not yet available to the general adult population in Mozambique through the national immunization program.

Consistent with other studies among PWID in surrounding countries [26–28], heroin was the most commonly injected drug among those who participated in our study (81.3% in Maputo and 73.0% in Nampula/Nacala). Also consistent with the drug trafficking history in the region, an expectedly high proportion of PWID (approximately one-quarter) initiated drug injection use during the 10 years prior to the survey. Not surprisingly, we found that PWID with a longer history of injection drug use had greater odds of HIV infection [29, 30], likely a result of longer exposure to injection-related risk over time. In addition, 10 years prior to the survey awareness of the modes of HIV transmission among Mozambicans was low [31].

Despite the fact that the majority of participants reported having access to new or sterile syringes, close to half reporting having ever shared syringes, and about one in ten used a needle or syringe after someone else had used it the last time they injected drugs. This inconsistency of sterile needle availability versus sterile needle use can potentially be explained by practices that are perceived by PWID to reduce drug waste, including reuse of their own needles, backload (a method of sharing drugs between needles), flash blood (a method of sharing blood of someone who injected) and sale of drugs in pre-loaded syringes. These practices have been identified in prior studies conducted in sub-Saharan Africa [32], including in neighboring Tanzania [7]; however, further investigation into the barriers to new, sterile syringe use in the Mozambican context is needed. Interestingly, not having access to new and unused syringes appeared to be a protective factor of HIV among PWID. While this may seem counterintuitive, we believe this is likely because persons who knew they were at high-risk for having HIV or already knew their HIV status were more likely to have received prevention messages and would know where to obtain sterile equipment. Nonetheless, the finding of high access to new and unused syringes but low utilization provides evidence for the importance of targeted harm reduction interventions.

Although transmission of HIV among PWID is mostly linked to injection drug use, sexual behaviors among PWID should also be considered important in countries generalized epidemics, such as Mozambique [33, 34]. Injection of stimulants, such as cocaine and methamphetamine, have been linked to high-risk sexual behavior [35, 36]. Among sexually active PWID (72.8%) in our study, approximately half in Maputo and nearly one in three in Nampula reported not using a condom the last time they had sex. Moreover, self-reported symptoms of Sexual Transmitted Infections (STIs) were high among PWID, reaching 28.6% in Nampula/Nacala. Sexual transmission of HIV might play an especially important role among PWID in Nampula, given that prevalence of HCV, which generally is not transmitted sexually, was lower than often found among PWID.

HIV transmission among PWID and their partners is compounded by the sizeable proportion of HIV-positive PWID who are not aware of their serostatus, who share needles, and who do not use condoms [37]. Our findings underscore the need for introducing harm reduction programs for PWID in Mozambique, as has been done in surrounding countries [19, 38], and especially in Maputo where the proportion of PWID having ever received any harm reduction or HIV prevention service was unacceptably low, even in comparison to Nampula.

Our study has some important limitations. Although RDS diagnostics revealed that the sample met the underlying assumptions needed for RDS analysis as presented in the full survey report [21] and that recruitment equilibrium was achieved for key variables, the Nampula/Nacala study site did not reach the desired sample size. This small sample size limits the confidence around estimates and reduces the ability to make significant comparisons between groups. There are several factors that could have contributed to the survey not achieving sample size in Nampula, the first is that the pool to sample from is small, estimated at 510 [21]. Secondly, police activity around drug use was high in the area. This activity was unrelated to the survey, but according to community outreach workers they hampered PWID willingness to participate. Another important limitation is that certain sub-groups of PWID were not captured; most notably females who inject drugs and non-Mozambicans. Though community outreach workers made substantial effort to reach out to females who inject drugs in their social networks, we had known from formative assessment that fewer females than males inject drugs, and that female injection drug users tend to be very difficult to access. This difficulty in recruiting female inject drug users is not unique to our study [7, 39, 40]. Our findings are also limited by the inclusion of individuals who reported lifetime, as opposed to recent, use of injection drugs, a necessary action to overcome bottlenecks in participant recruitment. Analysis of key variables pre- and post- exclusion criteria change found no difference between participant composition. HIV prevalence based on self-reports likely underestimate true HIV prevalence because persons with HIV are likely reluctant to disclose status due to stigma. The high refusal rate for hepatitis C testing likely underestimates the true prevalence of anti-HCV positivity in this population. Refusal for hepatitis C testing might be related to the lack of specific of the point-of-care test used, or the lack of hepatitis C treatment availability in the country. If treatment is introduced in the future, the proportion accepting hepatitis C testing may change [41]. Finally, though interviewers were highly trained and fluent in Portuguese and local languages, face-to-face interviewing methods, such as those used in this BBS, are prone to social desirability bias. Despite these limitations, the results presented here provide valuable insight into HIV risk behaviors among PWID in Mozambique that can guide targeted harm reduction interventions and policies.

## Conclusions

This was the first survey to document of the high prevalence of HIV, HBsAg and anti-HCV positivity among PWID in the Mozambique. Our findings suggest immediacy is needed in implementation of HIV, HBV and HCV prevention, care and treatment services for PWID. Additional studies may be useful to assess the feasibility of evidence-based harm reduction interventions such as needle and syringe exchange programs or opioid substitution therapies in Mozambique.

## Data Availability

The questionnaire used in the survey have previously been published and are available in survey Report [21]. The dataset generated and/or analyzed during the current study are fully available at the Mozambique National Institute of Health (INS) data repository for researchers who meet the criteria for access to confidential data. Datasets are from the BBS Mozambique study’s whose authors may be contacted through: www.ins.gov.mz.

## Abbreviations

Anti-HCV: Hepatitis C virus antibody
BBS: Biological and behavioral survey
HBsAg: Hepatitis B surface antigen
HBV: Hepatitis B virus
HCV: Hepatitis C virus
HIV: Human immunodeficiency virus
PWID: People who inject drugs
RDS: Respondent-driven sampling
